# Process-Induced Changes in the Quantity and Characteristics of Grain Dietary Fiber

**DOI:** 10.3390/foods10112566

**Published:** 2021-10-25

**Authors:** Ndegwa H. Maina, Anne Rieder, Yamina De Bondt, Noora Mäkelä-Salmi, Stefan Sahlstrøm, Outi Mattila, Lisa M. Lamothe, Laura Nyström, Christophe M. Courtin, Kati Katina, Kaisa Poutanen

**Affiliations:** 1 Department of Food and Nutrition, University of Helsinki, P.O. Box 66, FI-00014 Helsinki, Finland; noora.makela-salmi@helsinki.fi (N.M.-S.); kati.katina@helsinki.fi (K.K.); 2 Nofima, Norwegian Institute of Food, Fisheries and Aquaculture Research, PB 210, N-1431 Ås, Norway; anne.rieder@nofima.no (A.R.); stefan.sahlstrom@nofima.no (S.S.); 3 Laboratory of Food Chemistry and Biochemistry, KU Leuven, 3001 Leuven, Belgium; yamina.debondt@kuleuven.be (Y.D.B.); christophe.courtin@kuleuven.be (C.M.C.); 4 VTT Technical Research Centre of Finland Ltd., P.O. Box 1000, FI-02044 Espoo, Finland; Outi.Mattila@vtt.fi (O.M.); kaisa.poutanen@vtt.fi (K.P.); 5 Nestlé Institute of Materials Science, Nestlé Research, Société des Produits Nestlé S.A. Route du Jorat 57, 1000 Lausanne 26, 1800 Vevey, Switzerland; Lisa.Lamothe@rdls.nestle.com; 6 Institute of Food, Nutrition and Health, ETH Zurich, Schmelzbergstrasse 9, 8092 Zurich, Switzerland; laura.nystroem@hest.ethz.ch

**Keywords:** dietary fiber arabinoxylans, beta-glucan, fructans, resistant starch, processing

## Abstract

Daily use of wholegrain foods is generally recommended due to strong epidemiological evidence of reduced risk of chronic diseases. Cereal grains, especially the bran part, have a high content of dietary fiber (DF). Cereal DF is an umbrella concept of heterogeneous polysaccharides of variable chemical composition and molecular weight, which are combined in a complex network in cereal cell walls. Cereal DF and its distinct components influence food digestion throughout the gastrointestinal tract and influence nutrient absorption and other physiological reactions. After repeated consumption of especially whole grain cereal foods, these effects manifest in well-demonstrated health benefits. As cereal DF is always consumed in the form of processed cereal food, it is important to know the effects of processing on DF to understand, safeguard and maximize these health effects. Endogenous and microbial enzymes, heat and mechanical energy during germination, fermentation, baking and extrusion destructurize the food and DF matrix and affect the quantity and properties of grain DF components: arabinoxylans (AX), beta-glucans, fructans and resistant starch (RS). Depolymerization is the most common change, leading to solubilization and loss of viscosity of DF polymers, which influences postprandial responses to food. Extensive hydrolysis may also remove oligosaccharides and change the colonic fermentability of DF. On the other hand, aggregation may also occur, leading to an increased amount of insoluble DF and the formation of RS. To understand the structure–function relationship of DF and to develop foods with targeted physiological benefits, it is important to invest in thorough characterization of DF present in processed cereal foods. Such understanding also demands collaborative work between food and nutritional sciences.

## 1. Introduction

Grains are the main crop and the most common staple food for populations around the globe [1]. Beyond providing a significant portion of basic nutrients such as starch and protein in the diet, they also are the major contributor to the intake of dietary fiber (DF) [2]. Cereal DF has unique features among all fibers due to the complexity of the cell wall architecture of the grain matrix and, accordingly, grains are the recommended sources of DF in our diet [3]. Cereal DF is considered beneficial to health, and higher intakes are associated with decreased risk of chronic diseases [4]. Furthermore, whole grains and cereal DF consumption is inversely associated with mortality, grain DF being one of the key protective components [3,5]. In a systematic review and meta-analysis regarding DF type and blood pressure, β-glucan intake was associated with reduced blood pressure [6]. The European Prospective Investigation into Cancer and Nutrition (EPIC) showed a statistically significant 11% decrease in colorectal cancer risk per 10 grams/day cereal DF intake [7]. DF from cereals, but not fruit and vegetables, was associated with decreased rectal cancer risk. Furthermore, the recent review of Prasadi and Joye (2020) [8] summarized that people who consume a higher number of servings of whole grain foods are at lower risk of developing coronary heart diseases, diabetes, obesity and certain gastrointestinal disorders.

The DF concept and definition have been subject to intensive discussion and debate for more than 60 years now [9]. The definition of Trowell (1976) [10] stated DF to be polysaccharides and lignin in plants that are resistant to hydrolysis by the digestive enzymes of humans. As DF is a nutritional concept, it consists of a range of compounds of varying structural diversity. The Codex Committee on Nutrition and Foods for Special Dietary Uses (2009) defines DF to include “carbohydrate polymers with three or more monomeric units, not hydrolyzed by the endogenous enzymes in the small intestine of humans and belonging to the following categories: (1) edible carbohydrate polymers naturally occurring in the food as consumed; (2) carbohydrate polymers, which have been obtained from food raw material by physical, enzymatic or chemical means and which have been shown to have a physiological effect of benefit to health as demonstrated by generally accepted scientific evidence to competent authorities, and (3) synthetic carbohydrate polymers which have been shown to have a physiological effect of benefit to health as demonstrated by generally accepted scientific evidence to competent authorities”. Cereal DF, the topic of this paper, falls in the first two categories depending on the processing and fractionation treatments performed. Recently, a consensus group [9] proposed that it may be useful to distinguish DF originating from plant cell walls that are part of the plant matrix included in a product from purified DF supplements that are added to a product for a specific health benefit. Both the intrinsic/natural DF and added DF supplements may be modified during processing and may not have the same physiological and metabolic effects of the native DF. It is thus a challenge for the food industry to produce palatable foods optimized also in terms of DF functionality.

Processing is a prerequisite for the use of cereal grains in food. Processing involves the use of one or more-unit operations, involving application of mechanical or thermal energy, hydration and often activation of the endogenous biological enzymatic system of the grains. These operations induce various macroscopic to molecular level changes in the grain components, hence affecting the nutritional, technological and sensory properties of the products [11,12,13,14]. While fractionation causes an uneven distribution of DF both in terms of quantity and quality in resulting fractions, additional changes occur in the DF matrix during further processing even without changes in proximate composition. While it is obvious that the physiological responses and technological properties of cereal DF depend both on botanical origin and processing history, research is still needed in order to develop reliable and valid mechanistic knowledge of the health benefits of DF [15]. As Poutanen et al. (2018) [15] emphasize, the major issue in published literature is that the source and properties of DF used in human trials are poorly characterized or described, therefore not allowing to reveal the true structure–function relationships of DF on health-related outcomes. Furthermore, the form in which DF is consumed (whole grain vs. isolated DF) and the specific processing methods and parameters used should be reported. Smith and Tucker (2011) [16] pointed out in their review of clinical trials studying the effects of DF on health that the effects of whole grain and isolated DF are not necessarily the same. In a recent study by Smith et al. (2020) [13], different processing technologies were shown to have very different outcomes of health-related properties of DF. Extrusion of whole wheat led to higher amounts of microbiota-accessible carbohydrates (MAC) but did not increase production of short-chain fatty acids (SCFA) in comparison to sourdough fermentation of the same wheat, which enhanced SCFA production but did not increase the amount of MAC.

**Figure 1:**
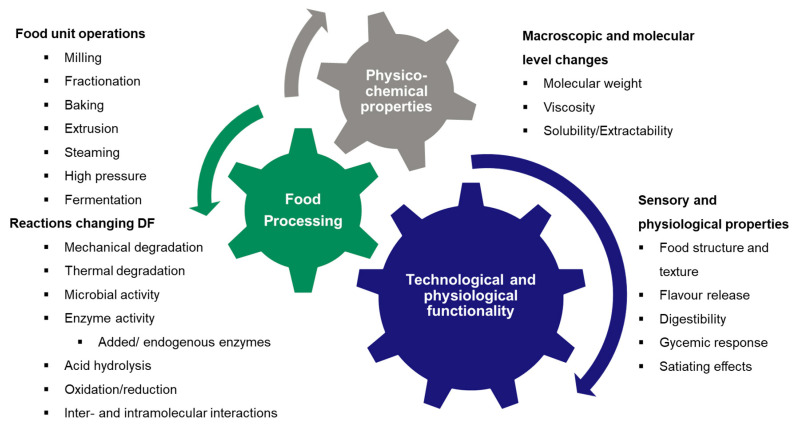
Overview of cereal processing operations and the changes in dietary fiber properties. The changes induced during processing can be intentional or unintentional and will either enhance or diminish technological and physiological functionality of dietary fiber.

While cereal processing (e.g., milling, extrusion, baking) is designed to create intentional changes in DF, unintentional changes due to oxidation and enzymatic hydrolysis by endogenous or microbial enzymes can also occur (Figure 1). Both intentional and unintentional changes in solubility, extractability and physicochemical properties of DF and restructuring of the grain matrix can either unlock or hamper the physiological and technological value of DF (Figure 1).

Several earlier studies have focused on the effects on DF components on sensory quality and consumer acceptability. The aim of the current review is to describe the effects of processing on the content and properties of cereal DF with respect to nutritional consequences. An overview of the structural features of DF components is first provided, followed by a review on their changes during processing. The challenge in assessing these changes is also briefly discussed. Finally, the physiological functionality of cereal DF in relation to processing-induced changes is featured.

## 2. Cereal Dietary Fiber Components and the Effects of Processing on Their Physicochemical Properties

The composition and structure of DF in cereal kernels depends on the type of cereal (Table 1). The total DF content of wheat, oat, barley and rye wholemeal is similar, between 10.6 and 25.2%. While in wheat and rye, arabinoxylan (AX) is the major DF, in oat and barley a significant amount of β-glucan is present. The highest fructan levels among cereal grains are found in rye (2.5–6.6%) and lower levels are present in wheat (<2.9%), barley (<1.0%) and oat (<0.2%). Other DF components found in cereal kernels include cellulose, lignin and resistant starch.

**Figure 2:**
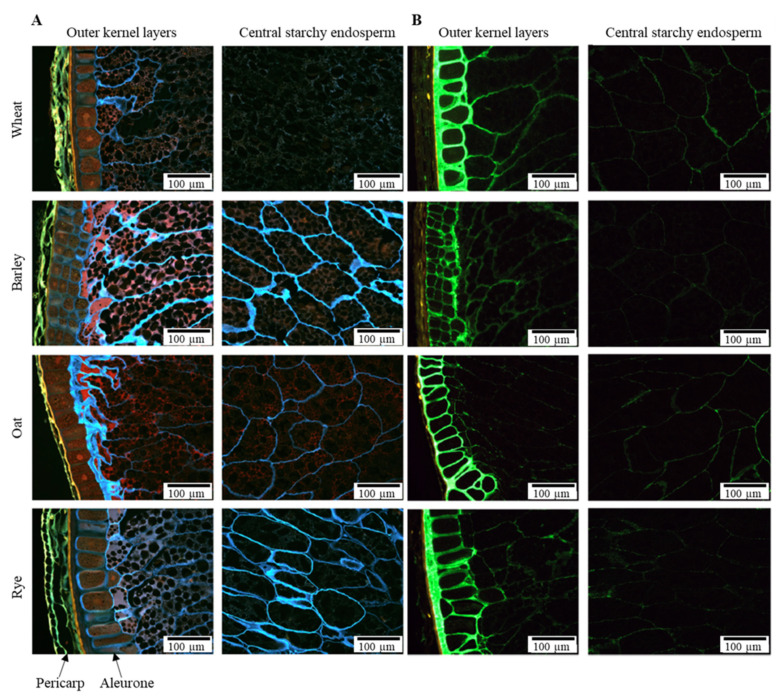
Epifluorescence microscopy pictures of outer kernel layers and central starchy endosperm of wheat, barley, oat and rye. (**A**) β-glucan is stained with Calcofluor (blue), protein with Acid Fuchsin (red). The pericarp is visible by autofluorescence (yellow). (**B**) Arabinoxylan is stained with an inactive fluorescently labelled xylanase (green) [35]. Adapted from Dornez et al. (2011) [35], reprinted with permission from Elsevier.

The DF content and composition also strongly depends on the anatomical part of the cereal. Cereal grains have a complex structure with different cell layers. In Figure 2, the microstructure of the outer kernel layers and the central starchy endosperm is shown for four types of grains. The differential staining of β-glucan and AX used in Figure 2 shows the differences in composition and structure of the DF in the cereal kernels [35]. The outermost pericarp layer is not stained in Figure 2B as AX is highly substituted in this layer and insensitive to the staining technique. The cells in the aleurone layer have much thicker cell walls than those in the starchy endosperm. Furthermore, a lamellar organization of AX and β-glucans has been observed in wheat (aleurone and endosperm cell walls) [36] and barley (endosperm cell walls) [37]. The different polymers of cereal cell walls are held together by non-covalent bonds and covalent crosslinkages. However, unravelling the 3D organization of cell wall polymers and other components within the cell wall matrix remains a challenge. The kernel cell wall polysaccharides (AX, cellulose and β-glucan) and lignin are the major contributors to DF in cereals, while the other DF components (fructan and resistant starch) are present within the cells. 

The spatial distribution of DF in cereals is also reflected in the DF composition of different milling streams. During milling, the outermost bran layers are separated from the endosperm fraction, which is reduced in particle size to a fine powder, the refined flour. The DF content of the refined flour (4.1–21.8%) is always lower than that of the bran (16.5–72.5%) for the four cereal types (Table 1). The large variation in DF content and composition in the milling fractions is a result of differences in milling procedures, cultivar, cultivation conditions and also DF analysis technique (see Section 3).

### 2.1. Arabinoxylans

In wheat, arabinoxylans (AX) constitute 4.0–9.0% of the kernel, which is similar to the AX level in barley (3.4–8.0%) and maize (5.1–6.8%), lower than that in rye (7.1–12.2%) but higher than the amount present in oat (2.2–4.1%) and rice (2.6%) [17,38,39]. AX are exclusively part of cereal cell walls [38,39]. In their most basic form, AX consist of a linear backbone of β-1,4-linked D-xylopyranosyl units, which can be substituted with monomeric α-L-arabinofuranosyl units at C(O)-2 and/or C(O)-3 positions [39]. The distribution of such arabinose units over the xylan backbone varies with cereal type. The degree of substitution (DS), expressed as the arabinose to xylose ratio (A/X), is also an important structural characteristic of AX. The AX of wheat, barley and rye, the pericarp, is characterized by a DS that exceeds 1.0, while the aleurone has a DS of 0.3–0.5 for wheat, 0.6–0.7 for barley and 0.4 for rye. The endosperm has an average DS between 0.5 and 0.9 for wheat, between 0.7 and 0.9 for barley, between 0.5 and 0.8 for rye and between 0.6 and 0.8 for rice [17,39,40,41,42,43]. In AX, phenolic acids such as ferulic acid can be esterified to the C(O)-5 position of arabinose. Such ferulic acid residues have the ability to crosslink and form diferulate bridges between AX molecules [44]. Other constituents such as glucuronic acid, its 4-O-methyl derivate or glucuronopyranosyl units can be bound to the C(O)-2 position of xylose [45,46].

The major part of AX is water-unextractable AX (WU-AX) due to covalent bonds or non-covalent interactions between individual AX-molecules or AX-molecules and other cell wall constituents [42,45,47]. Water-extractable AX (WE-AX) are rather loosely bound to the cell wall surface. Arabinoxylan-oligosaccharides (AXOS) are degradation products of AX. AX can have a great impact on several cereal-based processes and sensory and nutritional quality of end products, but the processes themselves can also change AX structures. Such changes can affect their physicochemical properties, which are inherent to their structural characteristics [39], impacting their technological and physiological functionality. 

#### 2.1.1. Milling and Fractionation

Grain kernels are most frequently milled in a roller mill process that combines breaking, reduction and sieving operations. At the end of the process, three main fraction types are obtained: flour fractions, mainly consisting of endosperm particles; bran fractions, strongly enriched in kernel outer layers; and germ [48]. The bran of wheat, barley, rye and oat is much richer in AX compared to the endosperm, and hence the flour. Milling of grain kernels can thus be used to separate AX-rich tissues from the tissues containing low amounts of AX. 

Another process frequently used to obtain different fractions from grains is pearling. Grain kernels are scrubbed against abrasive stones and against each other. This combination of abrasion and friction results in the removal of the outer layers of the grain kernels. The intensity of the pearling process determines which layers are removed [49,50]. For example, 10% removal of the grain by pearling has been shown to decrease the AX content by about 30% [51]. Thus, by pearling, different bran type fractions with varying AX contents and AX properties can be obtained [52]. 

Separation techniques such as sieving and electrostatic separation can be utilized to separate AX-rich tissues from other grain tissues after milling [53,54]. Since aleurone layer and endosperm fractions carry a positive charge, and pericarp, rich in AX, carry a negative charge, AX-rich fraction can be produced with electrostatic separation. With the combination of electrostatic separation and sieving, fractions with AX content up to 43% (dm) have been produced from finely milled wheat bran [53], although rather low yields were reported [53,54].

When bran is milled, changes to the AX population will depend on the severity of milling and the milling technique. Reducing the average particle size of wheat bran from around 1.5 mm to 77 µm with impact milling, resulted in an increase in WE-AX from 0.6 to 1.0% [55]. However, cryogenic milling until a median particle size of 6 µm increased the WE-AX content of wheat bran up to 2.8% [56] and, with very extensive planetary ball milling treatment, more than 17% WE-AX in the form of AXOS have been produced in situ [57].

#### 2.1.2. Baking Process

Majority of the changes in DF components during baking processes occur during the dough making and pre-fermentation. During straight dough breadmaking, without the use of added enzymes, changes in the AX population are relatively minor. Cleemput et al. (1997) [58] and Leys et al. (2016) [59] demonstrated that during dough mixing and fermentation, 5 to 20% of wheat flour AX population is solubilized. This was attributed either to the mechanical work input during mixing, the temperature increase during fermentation or the impact of endogenous and grain-associated microbial xylanases present in wheat flour [60,61,62]. 

Targeted solubilization of WU-AX with added xylanases during dough mixing and fermentation leads to a reduced water-binding capacity of AX and, therefore, redistribution of the previously bound water to other dough components [63]. Leys et al. (2020) [64] showed with 1H NMR that WU-AX withdraws water from other flour components during resting and that this effect could be reduced by the addition of xylanase. The amount of WU-AX solubilized by xylanases depends on the dosage of the enzyme used but can reach 90% when the excessive water release is compensated for. Solubilization of WU-AX to high molecular weight (HMW) WE-AX during bread making increases dough stability [65]. Analysis of the extensional viscosity of dough suggests that starch–starch and starch–gluten interactions are favoured by the increased water availability as a result of xylanase activity, and this drives the increased dough stability [59]. Another hypothesis is that the HMW WE-AX increases the viscosity of the dough aqueous phase and thus stabilizes the liquid films surrounding the gas cells which consequently slows down their coalescence [66,67]. This results in an increased loaf volume and a finer and more homogenous crumb structure. After baking, a part of the previously solubilized AX becomes unextractable again [59,68]. Hydrolysis of HMW WE-AX or soluble AX (S-AX) to low molecular weight (LMW) S-AX by excessive amounts of xylanases should be avoided from a processing point of view as it decreases both the water binding capacity and viscosity forming capacity of the AX fraction and results in slackening of the dough [47]. From a nutritional perspective, however, conversion of insoluble AX into S-AX and even AX oligosaccharides (AXOS) by xylanase can enhance the prebiotic properties of the food product [69,70]. Effects of AX on baking process and bread quality well demonstrate the dual role of cell wall polysaccharides in technological and nutritional functionality.

Sourdough fermentation has profound influence on the quality of AX. The most reported phenomenon is solubilization, which occurs during rye sourdough bread baking [71], wholegrain wheat fermentation [72] and wheat bran fermentations with and without added xylanases [73,74,75]. Depending on the fermentation type, molecular weight (MW) of AX can decrease [71] or remain unaffected [72]. Positive influence of sourdough fermentation on bread texture and volume have been attributed to the solubilization of AX [74,76].

##### Refrigerated Doughs

A common problem with refrigerated doughs is the development of a brownish fluid on the dough surface and in the package, also called syruping, after prolonged storage [77]. This is caused by endogenous flour xylanases which hydrolyze WU-AX to HMW S-AX during storage. When HMW S-AX are further degraded, their water-holding capacity decreases, their viscosity increasing capacity is lost and, therefore, syruping occurs [77,78]. The problem can be solved by selecting cereal varieties with a low level of endogenous and flour associated xylanases [79], by debranning the cereals before milling to reduce the xylanase load [80,81], by addition of xylanase inhibitors [78] or by addition of xylan as competitive substrate for AX [82]. These processing procedures therefore aim for refrigerated dough products with minimal changes to native AX structure and physicochemical properties.

#### 2.1.3. Germination

Besides for malting, germination is applied to improve the nutritional value of cereals and pulses and, in particular, of brown rice [83]. Rao and Muralikrishna (2007) [84] showed an increased yield of S-AX from rice, accompanied with an increased DS due to malting. This increased DS was explained by the activity of xylanases during germination, which primarily cleave unsubstituted regions of the xylan backbone, thereby solubilizing AX fractions having a high-branching degree. Rao and Muralikrishna (2007) [84] also showed that AX from malted rice had a high content of ferulic acid and the MW had a bimodal distribution (MW of 40,000 and 75,000 g/mol). Prior to malting, the MW of AX was 231,000 g/mol, thus further evidencing depolymerization during malting.

Singkhornart et al. (2014) [85] reported increased S-AX contents in germinated wheat when compared with the non-germinated one. Similar observations were made by De Backer et al. (2010) [86] who studied the xylanase activity and AX contents during different stages of germination. The study demonstrated that in the early stages of germination, the level of S-AX increased. In the intermediate stages of germination, xylanase activity increased and, therefore, more solubilization of WU-AX occurred. Simultaneously, the amount of WE-AX reached its maximum after which it decreased. 

#### 2.1.4. Extrusion

Extrusion cooking is a continuous process in which food material is cooked under high pressure, high temperature and high mechanical shear in a short time and is commonly used to produce expanded cereal product. The process results in various chemical reactions and the disruption of cell wall structures. Extrusion has been shown to increase extractability of wheat AX [87,88,89,90]. According to Andersson et al. (2017) [87], the wheat bran AX extractability increased from 5.8 to 9.0% and from 14.2 to 19.2% in rye bran. The study showed that the highest extractability of DF was observed when using high screw speed, high temperature and low water content. Demuth et al. (2020) [88] found similar results for wheat bran but also showed that A/X ratio slightly decreased, while MW was not significantly reduced (403,000 and 345,000 g/mol for native and extruded WEAX, respectively). Roye et al. (2020) [90] also showed that the DP of WE-AX remained high (DP112, A/X 0.72) after high shear and low moisture extrusion cooking compared to low shear, low moisture extrusion cooking (DP 58, A/X 0.81). In the study, the AX of the unextruded control had a DP of 45 and the A/X was 0.88, thus indicating the solubilization of WU-AX with less substitution. Dang and Vasanthan (2019) [91] also showed that extrusion treatment of rice bran increased the yield of S-AX (3.5–5%) compared to unextruded control (2%). The most significant increase was seen at high screw speed and high moisture content. It is likely that the high shear rates with increased screw speed cause disruption of covalent and non-covalent bonds leading to a MW decrease and thus more soluble AX fragments. Furthermore, ferulic acid sidechains are liberated and lignin is softened, causing redistribution of DF from the insoluble to the soluble fractions.

When sampling and analyzing the AX population of three industrial pasta processing lines for different products (macaroni, capellini and instant noodles), Ingelbrecht et al. (2001) [92] observed that the level of WE-AX increased from 0.4 to max 0.7% out of the total AX content of 2.6%. This was probably due to mechanical forces as only very low xylanase activities could be detected in the raw materials. The MW of WE-AX was slightly reduced. At optimal cooking times, WE-AX losses in the cooking water were small (max 5.9%), while overcooking led to more losses. In a model pasta extrusion process, Comino et al. (2016) [93] demonstrated an increase in WE-AX levels by 12–15% with wheat, rye and barley flours. There were no significant changes in A/X ratio in extruded materials of the same study.

#### 2.1.5. Nixtamalization

Maize is the predominant cereal in the diets of Mesoamericans. It is traditionally prepared by a process known as nixtamalization that consists of cooking maize grain in lime water and steeping it for 12 to 24 h. Alkaline cooking affects AX as feruloyl-ester linkages are cleaved and WU-AX is solubilized [94,95,96]. The steeping liquid itself, “nejayote”, is also rich in AX, around 19% of nejayote solids, with a DS of 0.57 [97]. Feruloylated AX from nejayote have a MW of 60 kDa which is significantly lower than the MW reported for alkali-extracted AX from maize bran or maize DF, which ranges from 200,000 to 500,000 g/mol [98,99]. 

As an environment-friendly option to nixtamalization, extrusion has been proposed as a technology that can be used to produce whole grain corn flours. Extruding maize flour prior to alkaline treatment increases the amount of S-AX [100]. However, extrusion alone does not directly solubilize AX or liberate their individual monosaccharide constituents. Platt-Lucero et al. (2013) [101] tested a combination of extrusion, lime and xylanase on whole grain maize flour and the impact of such process on the quality of maize dough. The results showed that the addition of xylanase and lime prior to extrusion of whole grain maize flour increased the amount of alkali-extractable AX, albeit of a lower MW due to the depolymerizing activity of xylanase. Such modifications to the AX result in maize flour with higher water absorption capacity and maize tortillas of higher flexibility and reduced firmness upon storage [101]. A summary of changes occurring in AX during processing is shown in Table 2.

### 2.2. Cellulose and Lignin 

Cellulose is the main structural component of plant cell walls and the most abundant organic polymer in nature. It is a linear and unbranched polymer consisting of up to 10,000 units of β(1→4) linked D-glucopyranosyl residues. Intramolecular hydrogen bonding between glucosyl residues in adjacent chains results in the formation of a strong crystalline microfibril structure, which does not dissolve in water. The cellulose microfibrils are crosslinked via hemicellulose molecules. For all cereals, cellulose is mainly located in the hull and outer parts of the kernel [104].

Lignin is a heterogenous and highly crosslinked phenolic polymer based on phenylpropane units. Lignin is the second most abundant polymer in nature and an important constituent of plant cell walls. It acts as nature’s “glue” in the cell wall and prevents water loss from plant vascular systems due to its hydrophobic properties and also protects plants from microbial attack because it is highly resistant to enzymatic degradation [105]. Even though lignin is not a carbohydrate, it is included in the definition of DF because it is intimately associated with DF polysaccharides and may also play an important role in the physiological functionality of DF. Similar to cellulose, lignin is located in the outer layers of the grain [41,106,107,108]. 

The impact of processing on cellulose and lignin in cereals has not received significant attention, probably due to their relatively inert nature and less significant technological and physiological role. Cellulose has mainly been studied in rye baking and wheat bran treatments, and also in barley and oat processing. Lignin is even less often included in the studies concerning the effects of processing on DF. One challenge is that typically cellulose and lignin are not analyzed by specific methods. Instead, they are reported, e.g., as constituents of insoluble DF, which also includes insoluble hemicellulose, or in the case of cellulose, as part of non-starch/ insoluble glucan, which also measures β-glucans and/or resistant starch. This makes it difficult to draw conclusions about the specific process-induced changes on cellulose or lignin.

#### 2.2.1. Milling and Fractionation

Cellulose and lignin are located in the outer layers of the grain and are removed in the milling process during the production of refined flours. There is only little data available on the content or properties of lignin during different cereal processes, except for the studies concerning chemical and physical fractionation methods for delignification and purification of interesting oligosaccharides from cereal side streams [109,110,111]. It is reported that some physical and chemical processes may create new crosslinks between lignin and other cell wall polymers, as reviewed by [107]. Production of lignin-rich fractions from brewer’s spent grain by different milling technologies, enzymatic fractionation and alkaline extraction was studied by Niemi (2016) [108].

#### 2.2.2. Baking Process

In baking, cellulose is considered a less reactive dough constituent than AX [112]. Boskov Hansen et al. (2002) [113] reported that the amount of bound glucose monomers in DF decreased during rye dough mixing and acidification, which could be explained by the breakdown of cellulose and/or β-glucans. In the study of Rakha et al. (2010) [33], DF was characterized in different rye products (crisp bread, soft breads and extruded products) and the differences in cellulose content were concluded to be mainly due to variations in ingredients, not processing conditions.

Cellulase enzymes are commonly used in baking to improve the properties of the end-product, and synergistic effects with other cell-wall hydrolyzing enzymes in baking have been reported [114]. However, without the measurement of the content and properties of cellulose itself, the specific effect of cellulase enzymes on cellulose polymers in baking remains unclear. In a study by Lambo et al. (2005) [115], fermentation with lactic acid bacteria was reported to decrease the content of cellulose during fermentation of oat and barley concentrate, probably due to the action of bacterial enzymes, but the authors conclude that the mechanism requires further investigation. 

#### 2.2.3. Extrusion

Some studies reported a reduction in the content of cellulose and lignin by extrusion. Dust et al. (2004) [116] studied the effect of extrusion conditions on chemical composition of barley grits, cornmeal, oat bran, soybean flour and hulls and wheat bran, and they reported a slightly decreased content of cellulose and lignin in the extruded samples. Hell et al. (2015) [109] showed that extrusion promoted the enzymatic accessibility of cellulose in the outer layers of wheat bran and released, overall, more glucose than untreated wheat bran. Among the different mechanical, chemical, enzymatic and fermentation pre-treatment methods studied by Hell et al. (2015) [109], the chemical pre-treatments showed, by far, the most pronounced effect regarding the enzymatic release of glucose from cellulose. Based on a study on corn DF, Singkhornart et al. (2013) [100] concluded that an extrusion process does not directly liberate any sugar but renders cellulose more amenable to attack by hydrolytic enzymes and reduction to simple sugars.

### 2.3. Beta-Glucan

Cereal β-glucan (mixed linkage (1→3, 1→4)-β-d-glucan) is a partially soluble DF, which is built primarily of β-1→4 bound cellotriosyl (DP3) and cellotetraosyl (DP4) units that are separated by β-1→4-linkages. Longer cello-oligosaccharide fragments (up to DP20), which decrease the solubility of the β-glucan, have also been identified. The fine structure of β-glucan is commonly characterized by the DP3/DP4 ratio, which affects various physicochemical properties of β-glucans, and is dependent on the source. Oat β-glucan commonly has a DP3/DP4 ratio of 1.5–2.3, whereas barley β-glucan contains relatively more DP3 segments (DP3/DP4 1.8–3.5). Amongst different grains, the content of β-glucan is the highest in oat and barley (3–11%) and generally below 1% in other grains such as wheat and rye. In the native grains, β-glucan MW is usually high and highest for oat (about 2 × 10^6^ g/mol), followed by barley (about 1.5 × 10^6^ g/mol), while rye and wheat β-glucan are of lower MW (about 1 × 10^6^ g/mol or lower) [117]. 

#### 2.3.1. Milling and Fractionation 

The commercial oat crop is predominantly the hulled type containing fibrous hulls that typically make up 25–30% of the total oat weight. Hulled barley varieties used commercially contain less hull (only 10–13%) compared to oat. Hulless barley varieties are available and they facilitate the use of barley for human food because of the reduced insoluble fiber in the hull. The first steps in barley dry fractionation are blocking (dehulling) and pearling, whereby the outer barley grain tissues are removed by an abrasive scouring action to produce an edible product for food. Blocking and pearling removes a total of 30–50% of the kernel. 

Knuckles et al. (1992) [118] tested different mills in combination with sieving to obtain fractions rich in β-glucan. The best process involves milling of dehulled barley by an abrasion mill and sieving the ground material through a series of sieves. The fraction with the highest β-glucan content had, depending on variety, between 18.6 and 22.5% β-glucan, most likely with minimal changes in β-glucan structure and MW. The yield of this fraction was, however, very low at 2.1–4.5%. Roller milling and hammer milling [119] have also been utilized before sieving, but no particular advantages compared to abrasion milling have been reported. Izydorczyk et al. (2014) [120] combined passages through four sets of corrugated rolls with sieving and usage of a bran finisher to obtain a DF rich fraction (DFRF) from barley with 18.1% β-glucan and a yield of 49%. When the DFRF was milled with a pin mill, sieved and passed through a bran finisher, the β-glucan content increased to 27.6% but the yield was reduced to 28.8%. Dry fractionation schemes combining pin milling and sieving (air classification) have been developed to produce coarse fractions enriched in β-glucan. Successful separation and concentration of β-glucan enriched fraction depends on parameters such as presence of hull, starch characteristics, fat content and particle sizes [121,122].

Various studies have achieved β-glucan fractions from dehulled, hulless and defatted barley with varying yields: a coarse fraction with 27.2% β-glucan with a yield of 9–10% [123], 17.7% β-glucan with a yield of 28% [124], 31.3% with a yield of 31% [122], 20.3% β-glucan with a yield of 16% [125], 23.5% with a yield of 9.4% [121] and 15.3% with a yield of 30% [126]. Heneen et al. (2009) [127] demonstrated that the lipids in oat endosperm are linked to protein and starch. Thus, the removal of lipids can enable more efficient separation of these components by dry fractionation, as shown by the higher β-glucan concentrations [124,125,128]. Sibakov et al. (2014) [129] obtained 40.3% β-glucan in the coarse fraction at a high yield (84.6%) using defatted non-heat treated oat bran as starting material. In pilot scale, after the second round of grinding and air classification, the concentration of β-glucan obtained from defatted oat was 31.2% and a yield of 8.8% [128]. These milling and fractionation processes have minimal effect on the properties of β-glucan but typically enhance its extractability.

Initial processing of oat involves a heat-moisture treatment called kilning that is conducted primarily to deactivate lipase that cause rancidity. Kilning and subsequent steaming and flaking also reduce oat endogenous β-glucanase activity to a very low level. Ames et al. (2015) [130], Andersson et al. (2004) [131] and Rieder et al. (2015) [132] demonstrated that β-glucan viscosity and MW were dramatically lower in extracts of raw oats than those of the corresponding kilned material. Depending on the production process, degradation of β-glucan is therefore expected to occur when raw oats are used as an ingredient in food production. 

#### 2.3.2. Baking Process

Compared to the β-glucan MW in barley and oat raw materials, baked goods such as bread [132,133,134,135], crisp bread [133,136] and cake/muffins [133,137] often contain β-glucans with a lower MW. The degradation of β-glucan occurs during mixing, fermentation and proofing and is caused by endogenous β-glucanases in cereal flour [131,132,138], but unaffected by the presence of yeast [131]. β-glucanases have been found in wheat, rye and barley flour [139,140,141] and a significant degradation of β-glucan occurred also in composite wheat breads prepared with oat or heat-treated barley flour [132,134]. β-glucan degradation is most pronounced during mixing, while heat treatment in the oven has no effect on β-glucan MW [131,132]. 

Strategies to retain β-glucan MW in bread aim at minimizing enzymatic degradation during processing. This can be achieved by adding the oat/barley ingredient at a later time point in the process, for example, after fermentation of the wheat flour dough [132]. The use of coarse oat or barley flour with large particles is also advantageous for the prevention of β-glucan degradation [131,132] due to slower hydration and solubilization of the β-glucan [134]. However, this may also decrease the solubility of β-glucan in the final product. 

Most studies investigating the effect of baking on the solubility of cereal β-glucan have shown an unchanged [135,136] or increased solubility [137,142] in the baked product (bread, muffin, crisp bread) compared to the raw material. This is, however, not a continuous process and the different processing steps during baking have different impacts on β-glucan extractability. Mixing increased the amount of extractable β-glucan in a rye based crisp bread with added oat bran and a composite wheat/barley bread [135,136]. Fermentation of the dough, on the other hand, resulted in a time dependent decrease in β-glucan extractability [135,136,138], which has been attributed to the formation of unextractable β-glucan aggregates [135]. The effect of heat treatment in the oven on β-glucan extractability varies greatly and has been reported to dramatically increase [142], moderately decrease [135] or have no effect [136] compared to the dough. This may be due to differences in the solubility of β-glucan in the doughs prior to baking, since low β-glucan solubility in dough has been shown to result in increased β-glucans extractability in breads [135,142]. Soluble β-glucans in the dough may easily form complexes with other macromolecules [143] which may result in decreased extractability. 

Baking has, in general, no effect on the molecular structure (cellotriosyl/cellotetraosyl ratio) of β-glucan or the amounts present [131], except for extensive degradation during long (20 h) sourdough fermentation of barley flour which has been shown to decrease the amount of β-glucan by 10–30% [144]. During sourdough fermentation of barley or oat, the MW of β-glucan decreases significantly, while β-glucan solubility increases [104]. The use of wheat flour sourdough (75 to 100% of all wheat flour as sourdough) in combination with oat bran has, on the other hand, been shown to increase β-glucan MW in the resulting bread compared to the use of unfermented wheat flour only [145], presumably due to a partial inactivation of β-glucan degrading enzymes in the wheat flour sourdough. 

Storage of baked goods has, in general, little effect on β-glucan MW, except when storage at room temperature is extended for more than 3 days [146], which is presumably due to microbial growth. Depending on the time and conditions of storage, storage may have a profound effect on β-glucan solubility. While frozen storage over 2–7 days had no effect on β-glucan solubility regardless of the freezing method [142,146], frozen storage for 1–2 months was reported to decrease β-glucan solubility in oat bran muffins [137]. Storage at room temperature may decrease β-glucan solubility, the extent of which, however, is quite variable. Some studies show a dramatic (50%) reduction in solubility upon 1 day storage, while others report only minor changes over 1 to 6 days [142,146]. 

#### 2.3.3. Extrusion 

Extrusion can have a negative impact on the availability of β-glucan as its extractability may be decreased due to the newly formed structures in the complex starch-based matrix. However, the extractability of β-glucan may also be increased when insoluble HMW β-glucans are converted to soluble β-glucans with lower MW. Additionally, extrusion can disrupt some of the cellular matrix and thus weaken the interactions between cell wall constituents which improves β-glucans extractability. Generally, minor or no changes have been observed in the contents of β-glucan in extruded oat and barley products [93,147,148]. Honců et al. (2016) [149] compared five barley varieties after extrusion and reported significant increases in β-glucan content (up to 0.5%) in varieties with normal starch, but not in waxy barley varieties. On the contrary, Chang et al. (2015) [150] indicated that both collet and cooking extrusion decreased the β-glucan content from 8.5 to approximately 7.5%, independent of the extrusion parameters, while Köksel et al. (2004) [151] showed that high shear configuration (but not low shear) of the extruder decreased β-glucan content, at greatest, from 4.8 to 3.6%. 

Sharma and Gujral (2013) [148] compared eight hulled barley varieties and reported that in all varieties the ratio of soluble to insoluble DF was increased from 0.7–1.5 to 1.2–3.1 and that extractability increased up to 8% as a result of extrusion. Comino et al. (2016) [92] compared the effect of extrusion on β-glucan from barley, wheat and rye and reported increases of 8, 22 and 14%, respectively, in extractable β-glucan. Gaosong and Vasanthan (2000) [147] studied the effect of barrel temperature (90–140 °C) and moisture content (20–50%) on the extractability of β-glucan from waxy and normal starch barley varieties and detected that, in both types of raw material, extractability was significantly improved by extrusion. In the waxy barley variety Candle, β-glucan extractability could be increased from 41.5% in the untreated sample up to 95.3% after extrusion. In the normal starch barley variety Phoenix, a significant increase from 26.8 up to 41.1% extractability was also observed but, overall, the extractability was significantly lower compared to the waxy barley. The higher the moisture content during extrusion, the higher the increase in extractability. In contrast, in the study by Brahma et al. (2016) [152] on extruded oats, extractability of β-glucan was shown to be improved at lower moisture contents (15–21%) compared to Gaosong and Vasanthan (2000) [147] (20–50%). 

Brahma et al. (2016) [152] further showed that no significant changes in the MW of oat β-glucan after extrusion were observed. Similarly, Saldanha do Carmo et al. (2019) [153] found a minor reduction in β-glucan MW with high energy input (145 Wh/kg), moisture content between 11.2 and 16% and die temperature of 174 °C. Tosh et al. (2010) [154], however, showed that a dramatic increase in oat bran β-glucan solubility (from 38.7 to 66.8–100% after extrusion), was accompanied by a decrease in MW (from 2,484,000 to 251,000 g/mol) with increasing energy input (highest 148 Wh/Kg) at 7% moisture content and die temperature of 237 °C. On the other hand, Honců et al. (2016) [149] showed an increase in the MW of barley β-glucan after extrusion. The increase could rather be a newly solubilized fraction of β-glucan that was insoluble prior to extrusion or the presence of aggregates in the analyzed samples. 

Collectively, these studies show that extrusion can significantly improve β-glucan extractability, but one needs to control the extrusion conditions to optimize the process. The optimal parameters are not the same for all grains, but rather need to be tailored according to the grain (oat vs. barley), variety (waxy vs. normal starch) and desired properties of the resulting extrudate. Changes in β-glucan MW are also likely dependent on the extrusion parameters.

#### 2.3.4. Other Thermal Treatments and Processing of Aqueous β-Glucan 

Thermal treatments alone, and not combined to other processes such as baking, milling or extrusion, also have an impact on the physicochemical properties of cereal β-glucan. Freezing and freeze-drying, for example, may affect the extractability of β-glucan depending on the product type and freezing method [146]. Beverages or other high moisture foods enriched with β-glucan are currently not readily available on the market as optimizing their stability remains a challenge to the food industry. Recent advances in understanding the factors affecting β-glucan stability in aqueous systems, however, will allow the control of β-glucan degradation also in beverages and other high moisture foods. Kivelä et al. (2012) [155] studied heating, high-pressure homogenization and ascorbic acid treatments of β-glucan extracts and demonstrated that all these processes caused decreases in viscosity of β-glucan solutions and were accompanied with the formation of oxidized functional groups (carbonyls) along the chain. Factors affecting β-glucan oxidation in thermal treatments and various reaction conditions have further been reported by [156,157]. These studies collectively show that β-glucan is prone to oxidation in aqueous systems. However, through controlling the storage conditions (especially prooxidative transition metals), it is possible to stabilize β-glucan also in solutions. A summary of changes occurring in β-glucan during processing is shown in Table 3.

### 2.4. Fructans 

Fructans are a group of carbohydrates which consist mainly of fructosyl units with either no or one glucose unit present in their chain. Fructans are synthesized by addition of fructose to sucrose, thus resulting in several alternatives in their core structure. Linking fructose to the fructose moiety of sucrose can result in either 1-kestotriose or 6-kestotriose as the fructan core structure, while addition of fructose to the glucose moiety of sucrose results in neokestotriose. The addition of β-(2,1) fructosyl units to the 1-kestotriose core results in inulin-type fructans, while addition of β-(2,6)-fructosyl units to the 6-kestotriose core results in levan-type fructans. Neo-inulin-type and neo-levan-type fructans contain a neokestotriose core and have internal glucose units, indicating that both fructose and glucose of the sucrose moiety are elongated with fructosyl units [160]. Graminan-type fructans are branched and contain a mixture of β-(2,1)- and β-(2,6)-linkages with a 1- or 6-kestotriose core and are the more abundant form in cereal grains. Wheat grains contain a complex mixture of fructans, including branched graminan-type and also neo-type fructans [21]. Oat flour was found to contain mainly inulin-type and neo-type fructans, with no graminan or levan-type fructans. Barley and rye flour mainly contain inulin-type and graminan-type fructans [160]. The degree of polymerization (DP) of fructans varies from oligomers to polymers and, thus, fructans can be divided into polymeric fructans and fructooligosaccharides (FOS). Compared to the other grain carbohydrates, the average DP in fructans is rather low. In wheat, most of the fructans have a DP of 3–5, and some polymeric forms with less than 20 units are present [19,21]. The total fructan contents in cereal grains vary from negligible amounts in maize, oat and barley to 0.9–2.3 and 3.6–6.6% in wheat and rye, respectively.

Fructans are rather easily degraded during food processing, which makes it somewhat challenging to produce products that rely on their functional properties. High temperatures and low pH can result in severe degradation. Additionally, processes including fermentation (such as bread making) may lead to severe fructan losses due to the microbial invertase or inulinase activity [161]. Fructans are easily solubilized in hot water, which causes leaching during cooking of fructan-containing food products [21]. 

#### 2.4.1. Milling and Fractionation

Although rye contains more fructans than wheat [21,162], wheat is the main cereal grain source of fructan due to the significantly higher consumption of wheat products throughout the world. Fructan content in cereals differs between the different milling fractions. The content is significantly lower in flour than in bran: 1.5 g/100 g and 3.7 g/100 g, respectively, for wheat [19], and 3 g/100 g and 7 g/100 g, respectively, for rye [163]. Nonetheless, the content of white wheat bread and wholemeal wheat bread are rather similar [162] due to the rather low bran content in the wholemeal bread. 

The extractability of fructans from grain material can be increased with extrusion or by using matrix-degrading enzymes such as xylanases [163]. Haskå et al. (2010) [164] fractionated wheat into a starch-fraction, a gluten-rich fraction and a water-extractable fraction with and without xylanase addition. Fructan content of both the gluten-rich and water-extractable fractions was shown to increase with the usage of xylanase to aid the extraction. Additionally, the portion of fructans extracted can be affected by the extraction conditions. Haskå et al. (2008) [19] studied the MW of the fructans extracted from wheat and showed that hot ethanol extraction resulted in larger average MW of fructans than the extraction with ethanol at room temperature. This implies that processing temperature may affect the solubility of different fructans; thus, in wet fractionation processes, the conditions might result in differences in the extracted fructans.

#### 2.4.2. Thermal Treatment and/or pH Changes 

Without harsh acidic conditions, inulin can tolerate mild heat treatments. In a study by Böhm et al. (2005) [165], dry heating at 100 °C showed no significant degradation of inulin, but above 135° degradation occurred. Moreover, Glibowski and Bukowska (2011) [166] did not observe significant degradation of inulin in solutions heated up to 100 °C when the pH was close to neutral. Similarly, pH, without being combined with elevated temperature, does not cause harsh degradation of fructans. Inulin-type fructans were shown to be rather stable towards low pH when heating was not involved [166]. However, an already slightly elevated temperature (40 °C) resulted in rather severe degradation when pH was 1. Even a slight increase in pH reduced the susceptibility to degradation, since at pH 3, significant degradation was shown only at 80 °C and above. 

FOS have been shown to be rather stable at elevated temperatures and at acidic conditions. Courtin et al. (2009) [167] showed a maximum of 10% degradation of FOS when heated (100 °C) in acidic or neutral conditions, although FOS were shown to be more susceptible to acidic conditions than the other tested oligosaccharides (AXOS) and xylooligosaccharides (XOS). In alkaline conditions (pH 11), FOS decomposed more drastically (about 40%) than in acidic conditions, although significantly less than XOS.

#### 2.4.3. Baking Process

In non-fermented breads, high fructan concentrations can be obtained [21]. Commonly utilized yeasts (such as Saccharomyces cerevisiae) have invertase (EC 3.2.1.26) activity, leading to partial degradation of fructan during dough mixing and fermentation [14]. Verspreet et al. (2013) [168] reported an almost 80% decrease in the fructan content of bread after baking with added yeast and the losses occurred during the mixing and fermentation processes, pointing towards the enzymatic activity rather than the heat treatment as the causal factor. The samples without yeast addition did not show similar fructan degradation. 

Degradation of fructans may be desired when producing food products with low fermentable oligosaccharides, monosaccharides, disaccharides and polyols (FODMAP). According to Li et al. (2020) [169], the activity of the yeast-produced invertase enzyme decreases when DP of fructans is increasing. Even more complete degradation of fructans has been reported with lactobacilli-originating extracellular fructosidase enzyme (EC 3.2.1.80). Li et al. (2020) [169] showed that fermentation of dough with fructosidase-producing lactobacilli resulted in rapid hydrolysis of fructans. Extensive fructan degradation was equally observed when using inulinase (EC 3.2.1.17)-secreting strains of Klyveromyces marxianus in wheat wholemeal bread and rye bread systems [161].

### 2.5. Resistant Starch

“Resistant Starch” (RS) is defined as “the starch and products of starch digestion that are not absorbed in the small intestine of healthy individuals” [170]. RS in foods has been classified into five distinct classes: (RS1) physically inaccessible starch, which is entrapped within whole or partly milled grains or seeds; (RS2) some types of raw starch granules and high-amylose (high-amylose corn) starches; (RS3) retrograded starch (either processed from unmodified starch or resulting from food processing applications); (RS4) starches that are chemically modified to obtain resistance to enzymatic digestion; (RS5) starches capable of forming complexes between amylose and long branch chains of amylopectin with lipids [171]. The amount of RS in food depends on several factors such as source, intrinsic properties of starch such crystallinity, amylose/amylopectin ratio, moisture, processing and storage time [172,173]. From the perspective of grain DF, mainly RS1, RS2 (cereals high in amylose content) and RS3 are relevant. 

#### 2.5.1. Milling and Fractionation

The extent of milling (i.e., particle size) influences starch digestion in cereals. In grains, starch is encapsulated in plant structure and, therefore, products with whole grain kernels are likely to contain more RS1 than flours. During milling, grain kernels are mechanically reduced to flour with desired particle size. The grinding process also damages starch crystalline regions making it more susceptible to enzymatic degradation. Large particles are digested more slowly since they have a smaller surface area compared to smaller particles [174]. Therefore, incorporation of whole grain coarse flour, broken kernels or whole kernels in a product leads to significantly lower glycemic responses. However, such products are seldom eaten raw and therefore the content of R1 in the consumed product is reduced depending on the food production process (e.g., heat and moisture content). Consequently, it is not possible to make a generalization on the content of RS in processed food based on the degree of milling.

#### 2.5.2. Thermal Treatment (Baking and Extrusion)

Thermal treatment leads to starch gelatinization which dramatically increases starch digestibility. This will significantly reduce R1 and R2 but can result in formation of R3. The degree of gelatinization will depend on the source of the flour, cooking parameters such as time, moisture content, temperature and cooling time. Liljeberg et al. (1996) [175] observed that changing from conventional baking (40 min, 200 °C) of pumpernickel bread to long-time low temperature baking (20 h, 120 °C) increased the content of RS from 3.0–6.6% (starch basis), the highest values obtained when lactic acid as added. When wholemeal rye was replaced with wholemeal high-amylose barley flour, RS increased to 7.7% (starch basis). In a follow-up study, Åkerberg et al. (1998) [176] studied the RS content in bread (70% whole grain) made from barley genotypes varying in amylose content (3–44%). The amount of RS (total starch basis) varied from <1% in waxy barley to approximately 4% in high amylose barley in conventional baking (45 min, 200 °C) and from 2 to 10%, respectively, in the long-time/low-temperature baking (20 h, 120 °C). 

Štěrbová et al. (2016) [177] studied the digestibility of starch in six standard wheat cultivars and one high-amylose wheat cultivar and found that on the overall, RS was lower is white flour (CWF) compared to wholemeal flour (WWF). RS content ranged from 1.8–16.9% in WMF and 0.1–6.6% CWF. The highest content of RS (16.9 and 6.6% in WMF and CWF, respectively) was in Australian amylose wheat (AAW, containing 50% amylose). Baking studies with CWF did not significantly influence the amount of RS in white bread and biscuits. However, the authors observed a higher variability in RS content in white bread, possibly resulting from differences in technological quality of the different wheat cultivars that can affect the bread crust to non-crust ratio. Due to the high amylose content, bread baked with AAW had the highest RS content of all cultivars. Nonetheless, the AAW bread had poor technological quality (high dough stickiness and low bread volume). 

Extrusion generally increases starch digestibility of extrudates [178]. Faraj et al. (2004) [179] showed extrusion cooking of waxy and regular pearled barley flour did not lead to significant formation of RS3 but observed a slight increase in RS3 when samples were refrigerated (4 °C for 24 h) before oven drying of the extruded flour. Kim et al. (2006) [180] found that for pastry wheat flour, RS increased with extrusion feed moisture content and storage time (4 °C, for 0, 7 or 14 days). However, screw speed had no significant effect on RS. These studies illustrate the need to not only optimize extrusion parameters but also post extrusion conditions such as freeze storage in order to maximize RS formation. 

Other processing conditions such as oil content and fermentation can affect the content of RS. Buddrick et al. (2015) [181] showed that addition of palm oil to wholemeal bread decreased the yields of RS due to amylose complexion with palm oil which interferes with amylose crystallization. Furthermore, the study showed that rye sourdough fermentation (24 h with a starter) had a great impact on RS formation compared to bulk fermentation of wheat or wheat/oat blends. This has been attributed to the increased organic acid content sourdough bread [181]. It should be noted that formation of RS from pure starch and flour (wholemeal or refined) may be different under similar conditions due to the presence of proteins, non-starch polysaccharides, etc., in flour, that may interfere with formation of RS [179], particularly RS3 and amylose-lipid complexes (RS5). 

## 3. Challenges in Assessing Process-Induced Changes in DF Properties

**Figure 3:**
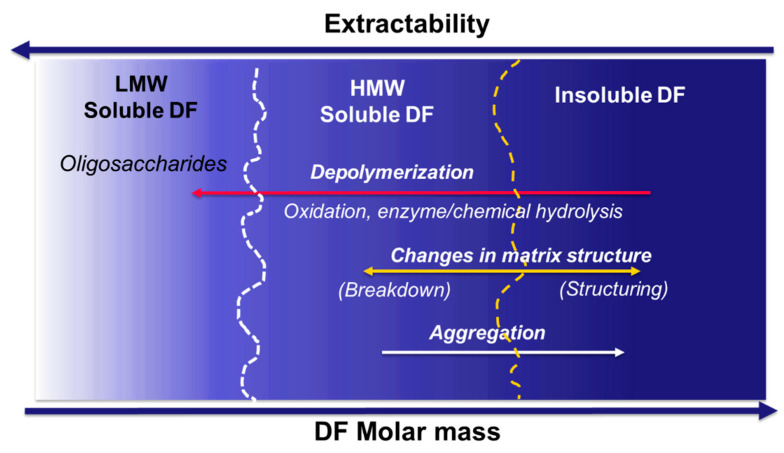
Changes occurring during food processing that lead to enhanced solubility/extractability of DF due to depolymerization or breakdown of the native food matrix or reduced solubility/extractability when the food structuring process leads to insoluble complexes (intra-molecular aggregates or inter-molecular complexes).

In their native state, DF are usually crosslinked (covalent and non-covalent linkages), forming a network that is not easily solubilized. In practice, the ratio of soluble to insoluble DF depends on the extraction conditions such as mechanical treatment, solvent, temperature and time. Consequently, comparison of data obtained from different studies using different processing parameters and analysis methods can be a challenge. Cereal processing can result in restructuring of the cereal matrix and depolymerization of DF, as summarized in Figure 3. It is, therefore, a challenge to determine whether changes in the ratio of soluble and insoluble DF fractions, for example, result from changes in the product matrix that enhances extractability and/or improved solubility due to depolymerization. Extractability or solubility, on the other hand, can also be reduced if the food structuring process leads to compact structures, entrapment, crosslinking or formation insoluble intermolecular/intramolecular complexes. 

A range of analytical methods have been used to evaluate process-induced changes in DF. Most studies have focused on changes in water extractability and solubility of specific DF during processing, in comparison to control untreated samples. Extraction, therefore, simply includes homogenization, dissolving the sample in water at a specific temperature and time period followed by centrifugations [78,182]. The extraction can also include amylase and protease treatment to remove starch and proteins, respectively. For RS analysis, Champ et al. (2003) [183] provide a review on the advantages and disadvantages of methods available. The important requirement is to control extractions conditions so that RS is maintained. At present, the most widely used method to study RS is based on the method of McCleary and Monaghan (2002) [184] (AOAC Official Method 2002.02; AACC Method 32–40.01).

Without further purification, the DF components in the extract are analyzed using colorimetric methods (pentosans), using specific enzyme kits from Megazyme (fructans, β-glucan, total starch and resistant starch) or with chromatographic methods after depolymerization (acid hydrolysis or methanolysis). The extracts have also been used to determine structural features of specific DF components, e.g., arabinoxylan A/X ratio after total monosaccharide composition analysis [41,185,186], β-glucan DP3/DP4 ratio using lichenase treatment and HPLC [187].

Determining the macromolecular properties of individual DF components entails sufficient purification. As an exception, calcofluor detection is considered to be selective for β-glucan and has, therefore, been used to determine the molar mass distribution of β-glucan in relatively crude extracts by coupling size exclusion chromatography with calcofluor detection [188]. The method, however, requires calibration with β-glucan MW standards, has been shown to exclude β-glucans with molar mass below 10 × 10^3^ g/mol [131,188,189] and has a tendency to underestimate weight average MWs over 500 × 10^3^ g/mol [188]. For samples with high MW β-glucan, such as native oat and barley samples, peak MWs could be used as these are not influenced by calcofluor detection [188]. For sufficiently purified DF components, it is preferable to determine the MW with absolute detection by using SEC or asymmetric flow field-flow fractionation (A4F) with refractive index (RI) and light scattering detection (LS) [190,191,192]. 

In vitro methods have been developed to provide data of DF in processed foods, allowing for prediction of postprandial glycemia and/or satiety. Several studies have shown a good correlation between the viscosity generated after in vitro digestion of the β-glucan containing test products and the extent of the physiological effect in humans [193]. A recent standardized comparison of published data indicates that the occurrence of coil overlap (corresponding to a critical zero shear viscosity in extracts after in vitro digestion) may be a prerequisite for the reduction in post-prandial glycemic response by β-glucan rich foods [194]. The dependence of certain health-promoting effects on the physicochemical features of cereal β-glucans and arabinoxylans indicate that it is extremely important for studies on process-induced changes on DF to simultaneously evaluate multiple factors including, total content, extractability, solubility, MW, viscosity and structural features (e.g., A/X ratio, DP3/DP4 ratio of β-glucan). Possibly, evaluation of these parameters under conditions that mimic the GI tract will provide a better correlation to health outcomes. Poutanen et al. (2018) [15] provide a comprehensive evaluation of the recommendations for characterization and reporting of dietary fibers in nutrition research. Investing in such thorough analysis and providing detailed information on the analysis methods will also facilitate comparison across different studies.

## 4. Physiological Functionality of Cereal DF in Relation to Processing-Induced Changes 

### 4.1. Cereal DF and Health

It is well documented that intake of cereal DF is inversely associated with the risk of chronic diseases, such as type 2 diabetes [195]. Such follow-up studies are based on questionnaires about food intake, and not much more about effects of cereal food processing can be concluded besides that the food consumed has been somehow processed. In intervention studies, however, effects of cereal food processing on various physiological functionalities have been addressed. Where DF has been considered, these studies have often suffered from limited analytical characterization of DF, which makes it difficult to make generic conclusions about the association of DF changes to physiological outcomes relevant for long-term health effects, as pointed out by [15].

DF influences physiological processes principally in two ways: by modulating digesta properties throughout the gastrointestinal tract from mouth to stomach and small intestine, and by influencing the extent and rate of microbial fermentation and formation of microbial metabolites in the large intestine. The process-induced changes of cereal DF components, described in previous sections, cause large variation in various intervention end points, such as gastric emptying, satiety, postprandial glycemic and insulin responses, blood cholesterol, inflammatory responses, transit time and formation of SCFA. These, in turn, are relevant for many studied long-term health outcomes.

For some DF sources, the relationship between their intake and specific markers of health outcomes has been sufficiently characterized to enable the authorization of health claims by EFSA. It is interesting to note that these claims are accepted in spite of the variation in DF characteristics due to effects of processing. The approved claims include: (1) consumption of rye DF contributes to normal bowel function [196]; (2) DF from wheat bran, oat or barley contribute to increased fecal bulk [197,198]; (3) consumption of at least 10 g per day of wheat bran DF in one or more servings contributes to reduction in intestinal transit time [198]; (4) consuming 8 g of AX-rich DF from wheat endosperm (at least 60% arabinoxylan by weight) per 100 g of available carbohydrates, or 4 g of β-glucans from oats or barley for each 30 g of available carbohydrates contributes to the reduction in the glucose rise after a meal [199,200]; (5) consumption of at least 3 g of oat or barley β-glucans per day contributes to lowering of blood LDL-cholesterol concentration [199,201,202].

### 4.2. Effects of Cereal DF Characteristics on Postprandial Events

The major physiological activity of β-glucan has been attributed to increased viscosity in the upper gastrointestinal tract, which is determined by extractability, solubilization and MW of β-glucan during digestion [194]. Increased viscosity results in delayed gastric emptying, alteration of gut hormone release, delayed starch digestion and delayed glucose uptake [203,204], explaining the reduced postprandial glycemic effect. Due to high viscosity forming capacity, WE-AX also delays gastric emptying which impacts the regulation of postprandial blood sugar and insulin levels. This means that processes increasing solubility and retaining high molecular weight of β-glucans and WE-AX have positive effects on postprandial glucose regulation and those causing extensive depolymerization diminish postprandial effects.

DF solubilization and depolymerization have a large effect on viscosity of both food matrix and food digesta. Increased solubilization of polymeric AX and β-glucans could theoretically increase gastric and ileal viscosity and retard gastric emptying and nutrient absorption. However, most often, solubilization during processing is accompanied by depolymerization, causing loss of viscosity, and, hence, reduction in the ability of, e.g., retarding postprandial glycemia [205]. This has been demonstrated numerous times, especially for oat β-glucans. Already in 1994, Wood et al. (1994) [206] showed a highly significant linear relationship between logarithm of viscosity of aqueous oat β-glucans solutions and glucose and insulin responses. This is also true for solid oat food matrices containing β-glucans, where the viscosity of subsequent food digestas depends both on hydration and the molecular weight of β-glucans [205]. Food storage and preparation may also influence DF extractability. 

### 4.3. Effects of Cereal DF Characteristics on Colonic Fermentation

The other important target of DF is the intestinal microbial fermentation. By definition, DF needs to be partly fermentable. Unextractable DF, such as WU-AX, increases fecal bulk and softens stool, partly due to high water binding capacity, and also shortens transit time. Fermentability of DF components depends on their chemical structure, degree of polymerization but also on matrix effects, meaning that processing can largely influence fermentability. For example, feruloylated arabinoxylans are less fermentable than their hydrolysis products AXOS. The SCFA formed as end-products of fermentation have various physiological effects, both in colon and peripherally, and their amount and site of formation depend on fermentability.

Another functionality gaining a lot of research interest is the ability of different DF polymers and oligosaccharides to selectively promote the growth of colonic bacteria. AX and AXOS thereof have been shown to produce butyrate and propionate and also to be selective, e.g., in modulating the ratio of Bacteroidetes to Firmicutes, with a suggested role in obesity, as reviewed recently by [207]. Feruloylated AXOS, formed by enzymatic hydrolysis of cereal bran during processing, also possess antioxidant properties, and the liberation and metabolism of ferulic acid in the colonic fermentation depends on the degree of hydrolysis of xylan during processing prior to consumption [208]. On the other hand, low molecular weight oligosaccharides can also cause gastrointestinal symptoms, especially in sensitive persons, such as irritable bowel syndrome (IBS) patients, and are therefore often classified as FODMAP compounds (fermentable oligosaccharides, monosaccharides, disaccharides and polyols [209]. Especially fructans but also xylo-oligosaccharides have been a concern here. In addition to choice of raw material, processing influences the amount of readily fermentable oligosaccharides and, hence, potential gastrointestinal symptoms. A specific sourdough process developed to deliver low-FODMAP bread is an example of use of processing for selective removal of certain DF compounds from a cereal food to make it more suitable in the diet of IBS patients [210]

Depolymerization and enzymatic modification of AX, β-glucans but also fructans may thus have a large influence on gut functions of cereal DF. Solubilization by mechanical, thermal, fermentation or enzymatic processing most typically increases in vitro colonic fermentation rate of cereal DF [211]. Moreover, particle size reduction in insoluble DF matrix has been shown to increase the fermentation rate [212]. Interestingly, a recent study demonstrated that only gut microbiomes that exhibited high carbon utilization responded in vitro to the effects of whole grain wheat processing and showed significant differences among processing methods. In these microbiomes, extrusion was able to increase microbial accessibility of the cell wall polysaccharides and sourdough fermentation led to increased production of butyrate [13].

Impact of β-glucan type on gut microbiota and host metabolism is still poorly understood. High MW β-glucan has been shown to modulate the gut microbiota differently than low MW β-glucan and promote bacteria that negatively correlate to CVD risk factors, e.g., bacteroides [213]. However, whether this is a direct consequence of promoting bacteria with a better ability to degrade longer β-glucans, as suggested for species of Bacteroides, is not yet known. It could also be an indirect consequence of different behavior of high-molecular β-glucan in the upper gastrointestinal tract that may result in a higher bile acid pool in the colon, which, in turn, could promote bile acid resistant bacteria. 

## 5. Conclusions and Future Prospects 

Process-induced changes in cereal DF components have been elucidated both from technological and physiological viewpoints. Even though DF is a nutritional concept, DF components have an important role in technological functionality, a prerequisite for successful food processing and consumption. Cereal food processes have been developed to maximize technological functionality and product palatability. The most common changes in cereal processing include cell wall matrix disintegration and particle size reduction, accompanied by depolymerization of DF components, which increases solubility, decreases viscosity and increases microbial fermentability.

Viscosity is of well-known importance to the upper GI tract responses (glycemia, satiety, etc.) and, especially, impact of processing on β-glucans properties has been addressed, i.e., how to retain molecular weight high enough to deliver health outcomes depending on viscosity. Here, efforts are still needed to find an optimal balance between technical and nutritional quality of food and to control unintended degradation of β-glucans. AX also deliver important technological functionality, e.g., in baking, and their hydrolysis and interactions with β-glucans and contribution to viscosity in gastrointestinal conditions should be more considered.

Colonic fermentation is increasingly acknowledged as a central event in control of many physiological processes. The colonic food, a large part of which originates from cereal DF, has a key role in variation of microbiota type, metabolic activity and metabolites. The impact of processing on cereal DF should, in the future, be further studied in this respect in order to reach optimal fermentation patterns. In the future, special cereal DF preparations could even be developed to contribute to favor specific microbiomes and maintenance of resilient microbiota.

Different cereal DF constituents have been studied a lot in isolation. The complexity of cereal DF may, in fact, be one of its relevant properties, influencing many physiological targets to maintain healthy homeostasis. Therefore, a holistic outlook and systems level understanding should be aimed at in future research. In the future, more emphasis will be laid on food structure and the impact of processing on nutritional quality of foods. 

Creation of the concept of ultraprocessed foods has led to new concerns about the influence of food processing on nutritional quality of food. Even if the discussion about ultraprocessing is more about ingredient types used, it also urges to reveal the changes food processing has on product quality. Grains cannot be consumed without processing, and while DF should be retained in food, processing can reduce or enhance its physiological functionality. As the majority of the population lacks DF in their diet, developing cereal foods rich in cereal DF is an important target. Knowing more about the fate of DF components during processing will assist both in delivering more cereal DF in the diet globally and in optimizing DF physiological functionality.

Adequate standardized analytical methodologies are needed to enable the comparison of different studies and generate structure–function relationships. In vitro predictive methods assist in elucidating effects of processing on physiological responses to DF prior to laborious in vivo trials. Linking DF carbohydrate chemistry and technology with nutritional physiology requests for bridging of food and nutritional sciences.

## Figures and Tables

**Table 1:** Composition of dietary fiber (% dm) in wholemeal, refined flour and bran of different cereals. DF = dietary fiber, AX = arabinoxylan, nd = not determined.

		DF	AX	Cellulose	Lignin	β-Glucan	Fructan	Ref
Wheat	Wholemeal	11.5–18.3	4.0–9.0	1.2–1.6	0.7–3.3	0.5–1.0	0.7–2.9	[17,18,19,20,21]
	Refined flour	4.1–4.3	1.4–2.8	nd	0.2–0.5	nd	1.4–1.7	[18,19,21]
	Bran	35.7–55.5	13.2–33.0	9.0–14.0	3.0–10.0	1.0–3.0	3.0–4.0	[18,19,21,22,23,24]
Oat	Wholemeal	10.6–23.4	2.2–4.1	0.8–1.2	1.3–5.9	1.1–5.6	<0.2	[17,21,25,26]
	Refined flour	9.5–13.1	1.0–1.3	nd	nd	1.0–1.1	nd	[26,27]
	Bran	16.5–24.7	3.5–13.2	ca. 1.4	nd	5.4–8.4	ca. 0.3	[22,24,26]
Barley	Wholemeal	15.0–23.8	3.4–8.6	1.4–3.7	1.5–4.7	3.7–6.5	<1.0	[17,20,21,28]
	Refined flour	4.8–18.3	1.2–6.0	0.9–1.6	nd	2.4–7.5	0.7–2.5	[28,29,30,31]
	Bran	ca. 72.5	4.8–9.8	nd	nd	6.2–7.6	nd	[22,28,30]
Rye	Wholemeal	20.4–25.2	7.1–12.2	0.6–1.2	0.9–3.2	1.7–2.6	2.5–6.6	[17,20,21,32]
	Refined flour	11.8–21.8	3.1–9.3	nd	0.2–0.5	1.5–3.4	3.1–4.6	[21,32,33]
	Bran	33.5–47.5	12.1–25.1	2.6–6.5	3.0–4.5	2.9–5.3	5.0–7.7	[21,22,24,32,33,34]

**Table 2:** Process-induced changes in arabinoxylans.

Process	Total Amount	Amount of WUAX	A/X Ratio (WUAX)	Amount of WEAX	A/X Ratio (WEAX)	Mw	Reference
Milling *							[41,48,102]
Bran	↑	↑	↑	↑	↑	nd	
Perikarp	↑	↑	↑	↓	↑	nd	
Aleurone	↑	↑	↓	↑	↓	nd	
Endosperm	↓	↓	↑	↓	↑	nd	
Baking process	-	↓	-	↑	-	/- a	[59,93]
Sourdough Fermentation **	-	↓	nd	↑	nd	↓/- b	[71,72,73,74,76,103]
Extrusion Pasta making	- -		-	↑ ↑	-	- c	[92,93]
Germination	↑	↑	↓	-/↓	nd	↓ d	[84,86]

* In comparison to wholegrain, ** in comparison to starting flour, ↑ increase ↓ decrease, - no change, nd not determined, a, influenced by amount of added xylanases, b, rye sourdough and use of xylanases promotes degradation of AX, c, only few studies, d, brown rice.

**Table 3:** Process-induced changes in β-glucans.

Process	Total Amount	Amount (Soluble β-Glucans)	Viscosity	Mw	Reference
Dry fractionation *					[118,123,128,129]
Coarse fraction	↑	↑	nd	nd	
Defatted, ultragrinded coarse fraction	↑	↑	nd	-	
Abrasion milling and sieving	↑	nd	nd	nd	
Pin milling/air classification	↑	nd	nd	nd	
Kilning	-	↓	↑ a	↑ a	[158]
Baking process **	-	↑↓	↓	↓	[131,132,133,134,135,136,137]
Sourdough Fermentation **	↓	↑	↓	↓	[104,144]
Extrusion ***	↑↓	↑	nd	↑↓-	[93,148,152]
Cooking Porridge	-	↑	nd	-	[159]

↑ increase, ↓ decrease, - no change, nd not determined, * in comparison to native grain, ** in comparison to starting flour, *** depends on processing conditions and analysis method, a, changes due to inactivation of β-glucan degrading enzymes during kilning.
